# A rapidly enlarging painful neck mass in a 59‐year‐old woman

**DOI:** 10.1002/ccr3.2637

**Published:** 2020-01-24

**Authors:** Priti V. Nath, Mohamed Shakir, Thanh D. Hoang, April E. Bergeron, Babette C. Glistercarlson

**Affiliations:** ^1^ Division of Endocrinology Department of Medicine Walter Reed National Military Medical Center Bethesda MD USA; ^2^ Department of Pathology Walter Reed National Military Medical Center Bethesda MD USA

**Keywords:** anaplastic, cancer, fine‐needle aspiration, thyroid, undifferentiated

## Abstract

Primary care physicians and endocrinologists should be aware that neck masses with concerning features like, in this case, a delay in evaluation and treatment may dramatically shorten a patient's life.

## Graphical abstract

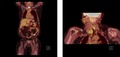

## 1 CLINICAL VIGNETTE

**Figure 1:**
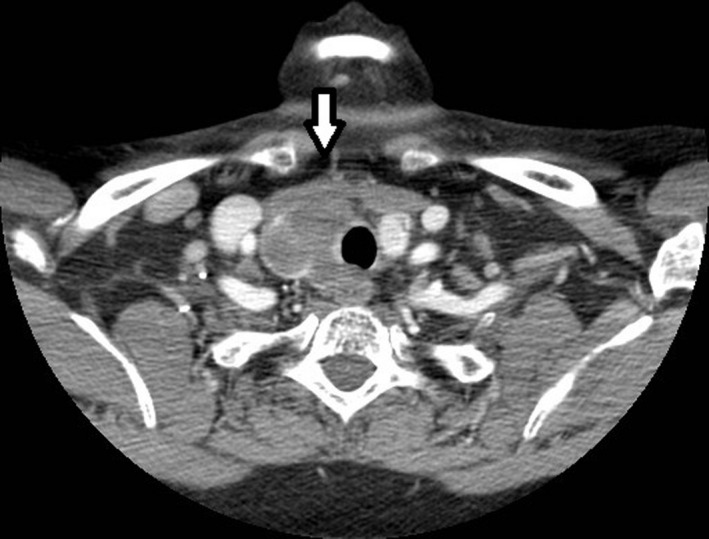
CT neck with solid‐cystic mass in the right thyroid lobe with variegated enhancement measuring 3.8 cm in largest diameter and no pathologic cervical lymph nodes

**Figure 2:**
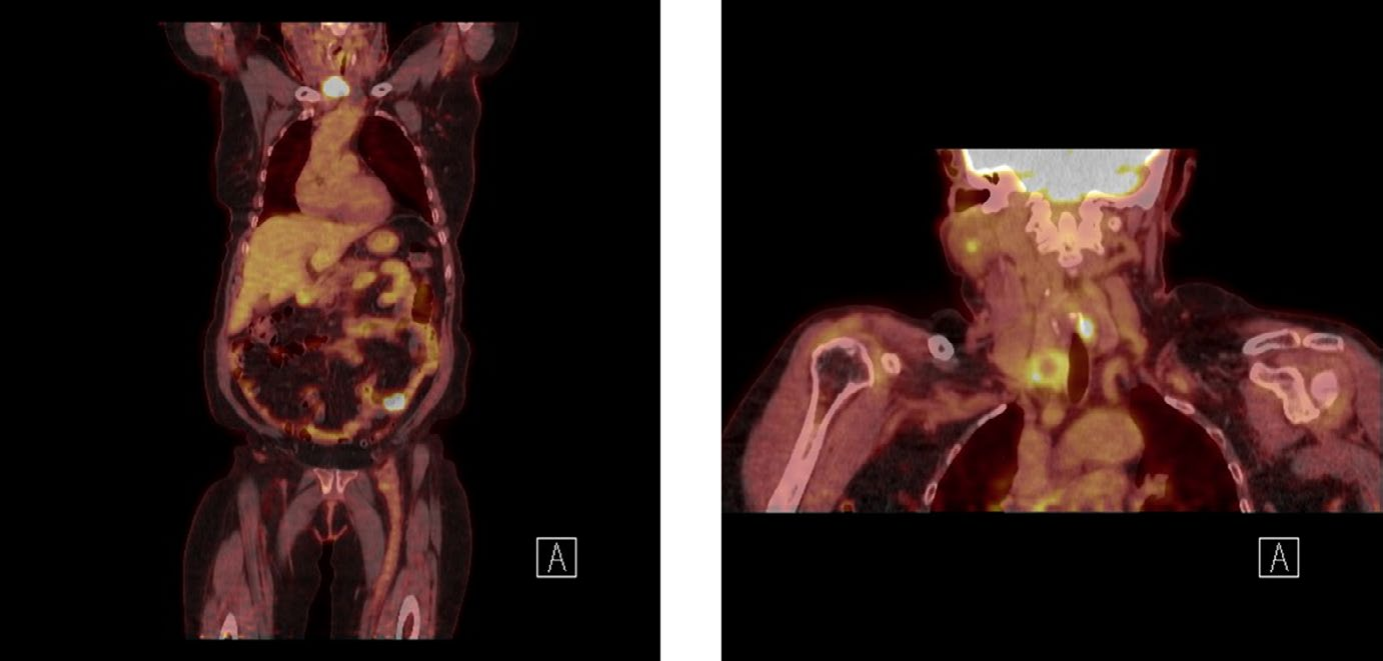
PET‐CT with distant disease within the small bowel (A) and lung (B) as well as inguinal lymphadenopathy (A)

A 59‐year‐old Kenyan female presented with 2‐month history of a right‐sided, enlarging, painful neck mass in the setting of headaches, hoarseness, dyspnea, and dysphagia. Examination revealed a 3.0‐cm hard, tender thyroid mass not fixed to overlying skin and the absence of cervical lymph adenopathy. Biopsy was consistent with dedifferentiated (anaplastic) thyroid cancer. Of note, this was the third biopsy attempt of the nodule. Two prior fine‐needle aspirations yielded only nondiagnostic, necrotic tissue. ThyroSeq Genomic Classifier demonstrated B‐Raf proto‐oncogene (BRAF) V600E negative, Tumor Protein 53 gene (TP53), phosphatase and tensin homolog gene (PTEN), neuroblastoma RAS viral oncogene (NRAS) positive malignancy. Diagnostic imaging was promptly obtained due to the aggressive nature of her disease. Computerized axial tomography scan (CT scan) of the neck (Figure 1) noted a solid‐cystic mass in the right thyroid lobe with variegated enhancement measuring 3.8 cm in largest diameter and no pathologic cervical lymph nodes. Magnetic resonance imaging (MRI) brain found a single dural‐based mass over the left occipital lobe with adjacent brain reaction. Positron emission tomography‐computed tomography (PET/CT) (Figure 2) found inguinal lymphadenopathy and distant disease within the lung and small bowel. Decision was made to surgically intervene. During the one‐month admission, patient underwent an uncomplicated total thyroidectomy, central neck dissection with tracheostomy placement and resection of the brain mass. In light of her distant metastatic disease and limited treatment options, she was given a dismal prognosis of 1‐6 months. After consideration, the patient decided to forego further treatment.

Anaplastic thyroid cancers are dedifferentiated tumors of the follicular epithelium. In contrast to differentiated thyroid cancers, they are rare and very aggressive. Despite the rarity (1‐2 cases per million), the disease is responsible for 14%‐50% of thyroid cancer mortality. One‐ and 10‐year survival estimates are 10%‐20% and <5%, respectively.^1^ There is no effective therapy for metastatic anaplastic thyroid cancer with few randomized control trials evaluating different strategies including a combination of surgery, chemotherapy, and radiation treatment.^2^ Doxorubicin has the highest overall response rate of approximately 20%.^3^ For patients with positive BRAF Valine (V) substituted by glutamic acid (E) at amino acid 600 (V600E) mutations, dabrafenib/trametinib treatment has recently shown an overall response rate of 69% in 11 of 16 patients in a small trial.

This case contributes to existing case reports of anaplastic thyroid cancer by underscoring the importance of early detection so that critical interventions, like tracheostomy placement, can be performed. Patients with complaints of a rapidly growing, hard neck mass warrant differential diagnoses including solid malignancies such as thyroid and oral‐pharyngeal cancers, lymphoma, reactive lymphoid processes, syphilis, leishmaniasis, and granulomatous diseases such as granulomatosis with polyangiitis and lymphomatoid granulomatosis. Benign lymphoid hyperplasia and aggressive differentiated thyroid carcinomas are also possible etiologies. As is noted in our case, prompt referral for biopsy is essential. Our patient had a delay in diagnosis as two prior fine‐needle aspirations were nondiagnostic. It has been postulated that fine‐needle aspiration can fail due to sampling in areas burdened with necrosis, fibrosis, or hemorrhage and the overall difficulty in obtaining adequate cells from masses containing both solid and cystic components.^1^ Primary care physicians and endocrinologists should be aware that neck masses with concerning features like, in this case, a delay in evaluation and treatment may dramatically shorten a patient's life.

## CONFLICT OF INTEREST

None declared.

## AUTHOR CONTRIBUTION

PVN: served as author. MS: served as reviewer. TDH: served as reviewer. April E. Bergeron, MD: served as pathology reviewer. Babette C. Glistercarlson, MD: served as reviewer.
